# Planned Neck Dissection Following Radiation Treatment for Head and Neck Malignancy

**DOI:** 10.1155/2012/954203

**Published:** 2012-09-24

**Authors:** J. F. Dautremont, M. K. Brake, G. Thompson, J. Trites, R. D. Hart, S. M. Taylor

**Affiliations:** ^1^Division of Otolaryngology-Head and Neck Surgery, University of Calgary, No. 511-505 19th Avenue SW, Calgary, AB, Canada T2S 0E4; ^2^Division of Otolaryngology, Dalhousie University, Halifax, Nova Scotia, Canada B3H 2Y9

## Abstract

*Introduction*. Optimal therapy for patients with metastatic neck disease remains controversial. Neck dissection following radiotherapy has traditionally been used to improve locoregional control. *Methods*. A retrospective review of 28 patients with node-positive head and neck malignancy treated with planned neck dissection following radiotherapy between January 2002 and December 2005 was performed to assess treatment outcomes. *Results*. Median interval to neck dissection was 9.6 weeks with a median number of 21 + 9 lymph nodes per specimen. Ten of 31 (32%) neck dissection specimens demonstrated evidence of residual carcinoma. Overall survival at two years was 85%; five-year overall survival was 65%. Concurrent chemotherapy did not impact the presence of residual neck disease. *Conclusion*. Based on the frequency of residual malignancy in the neck of patients treated with primary radiotherapy, a planned, postradiotherapy neck dissection should be strongly advocated for all patients with advanced-stage neck disease.

## 1. Introduction

 Metastatic neck lymph nodes are a common finding in the setting of head and neck cancer and serve as an important adverse prognostic factor in influencing management and patient survival. The management of these patients is generally guided by which modality is primarily used for treatment and whether a multimodality approach is required. For early disease (N0-N1), the common approach is a single-modality approach to both the primary site and neck disease, either surgery or radiation, with the trend towards radiation with the aim of organ preservation at the site of the primary malignancy [1]. In the N0/N1 neck, roughly equivalent rates of disease control have been found with radiation alone or in conjunction with planned post-radiotherapy neck dissection [2]. 

 More controversy exists in the management of the advanced head and neck cancer patient (N2-N3). Proponents of the multi-modality approach have presented the ideal of organ preservation through less intensive interventions while maintaining or improving survival. However, detecting the presence of locoregional recurrence and occult neck metastasis remains a challenge with this approach, with such recurrence representing failure of the initial treatment [2]. The two approaches used in response to this challenge are post-radiotherapy serial observation with salvage surgery for clinically detected neck disease and elective planned post-radiotherapy neck dissection (PPRND).

 Brizel et al. found that planned postradiotherapy neck dissection conferred an advantage in both disease-free survival and overall survival in patients with N2/N3 cancer, with acceptably low morbidity [3]. Elective PPRND done 4–12 weeks after radiotherapy has shown a relatively low complication rate and that 35% of specimens have residual disease which was otherwise clinically undetectable [4]. In looking at long-term outcomes, however, Argiris et al. found that planned postradiotherapy neck dissection had no impact on survival of N2 patients found to be clinically disease-free following radiotherapy [4].

 Discerning the role of planned postradiotherapy neck dissection in advanced head and neck cancer is critical to improving outcomes. The purpose of this study is to add to the current literature, better establish the value of planned after radiotherapy neck dissection, and clarify the recommendations for management of this patient population.

## 2. Materials and Methods

 All patients at our centre who had undergone radiation therapy for squamous cell carcinoma neck disease between January 2002 and December 2005 inclusive were reviewed for study eligibility. The patients within this population who had undergone a planned neck dissection following completion of radiation therapy to the neck for squamous cell carcinoma were included in the study. 

 The demographics collected included primary tumor site, TNM staging, and type of neck dissection performed. These patients were followed in terms of disease control—both locally and in the neck—and of absolute survival. Patients were staged using the AJCC 2010 TNM staging criteria, and survival curves obtained using the Kaplan-Meier statistical analysis [5]. Overall outcomes were presented as 5-year survival.

 Prior to commencing the project, ethics approval was obtained from the Capital Health Research Ethics Board in Halifax, NS, Canada.

## 3. Results and Analysis

 Twenty-eight patients met the inclusion criteria defined above. The mean age of the group was 56 ± 9 years and the mean follow-up period was 3 years. Twenty-six of the patients received continuous, full-course radiation therapy, and 19 of the patients underwent concurrent chemotherapy. 

 There were 31 neck dissections among the patient population; three patients underwent bilateral neck dissections and each side was counted as a separate neck dissection. The decision for bilateral neck dissection was based on clinical nodal staging after completion of radiation therapy and based on the primary site location. Although 6 patients had cN2c disease initially, only 3 of these had evidence of bilateral neck metastasis after radiation therapy requiring a bilateral neck dissection. The median interval from the end of radiation therapy until neck dissection was 9.6 weeks. The most common primary sites were the tonsil and tongue base, at 46% and 36%, respectively (Table 1). 

 All but 1 patient had stage III/IV disease (Table 2).

 Of neck dissections completed, 25 (81%) were selective dissections, 3 (10%) were modified radical dissections, and 3 (10%) were radical dissections. Selective neck dissections included levels II through IV. The median lymph nodes removed were 21 ± 9 (4–35). In total, 10 of the 31 (32%) neck dissections demonstrated evidence of residual carcinoma on pathology report (Table 3).

  

**Figure 1:**
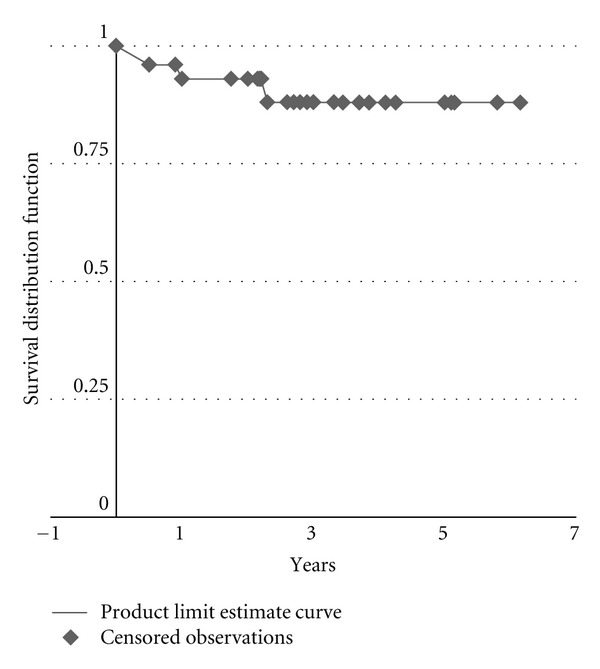
Primary site disease control in advanced head and neck cancer treated with postradiotherapy neck dissection.

 Overall primary site disease control in patients treated with postradiotherapy neck dissection was 93% at 1 year, 88% at 2 years, and 87% at 3 years (Figure 1).

**Figure 2:**
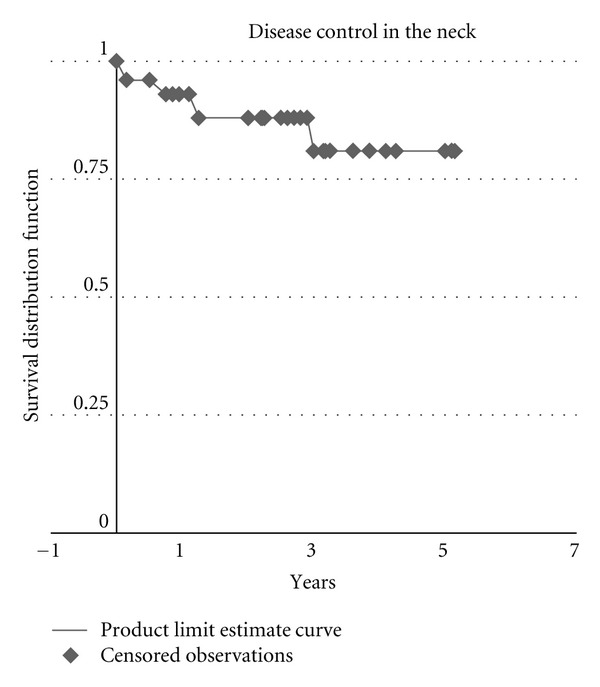
Disease control in the neck in advanced head and neck cancer treated with postradiotherapy neck dissection.

 Disease control in the neck of postdissection patients was 93% at 1 year, 88% at 2 years, and 81% at 3 years (Figure 2). 

**Figure 3:**
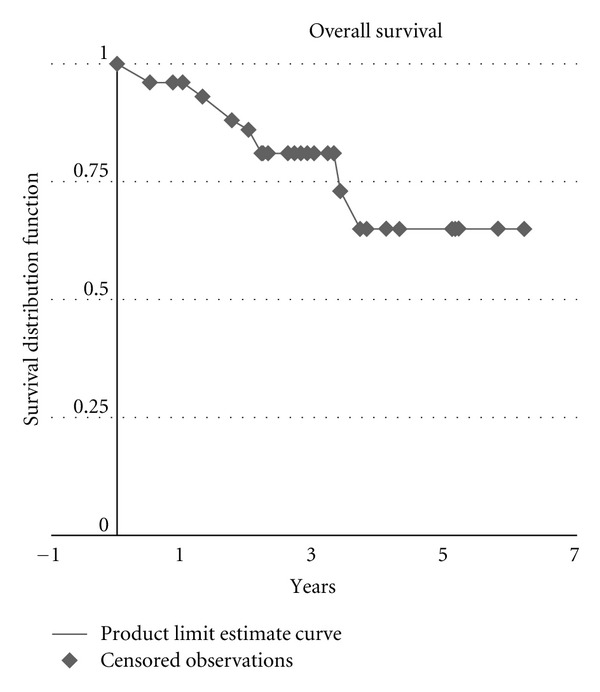
Overall survival in patients with advanced head and neck cancer treated with postradiotherapy neck dissection.

 Overall survival in the group was 88% at 1 year, 81% at 2 years, and 65% at 5 years (Figure 3). 

 When comparing overall outcomes to results of elective neck dissection pathology, of patients who had residual disease on pathology, 44% later failed in terms of neck control. This correlates to 14% of patients overall. Of the patients who died, 57% had known recurrent or persistent disease in the neck.

## 4. Discussion

 Advances in technique and management of side-effects have made radiotherapy a prominent modality in therapeutic planning for head and neck cancer. Radiotherapy has been shown to significantly reduce the yield of both total nodal yield during neck dissection, and the amount of positive nodal disease found. Combination chemoradiotherapy has also shown this benefit [6]. A significant amount of neck dissections, however, continue to yield positive nodal disease, despite these measures. Boyd et al. (1998) and Sewall et al. (2007) found no correlation between patient age, presenting T-stage, pretreatment nodal size, radiation dose, or type of neck dissection with the presence of carcinoma in dissection specimens [1, 2]. Another series, in fact, found neck metastasis to be present in one-fourth of dissections done in patients that completed cisplatinum-based chemoradiotherapy. The authors concluded that strong consideration for neck dissection in this group is warranted [7, 8]. 

The subject of much investigation has subsequently been directed towards guiding the use of postradiotherapy neck dissection. Several groups have focused on the role of clinical/radiographic response in influencing treatment planning. This has been done in an effort to discern which patients should be exposed to the potential morbidity of surgery. Some have advocated that clinical complete response is a valid indicator for omitting neck dissection in patients that can be reliably followed, citing neck failure rates of ≤5% in those with no evidence of neck disease on CT scan at 4–6 weeks following radiotherapy [9–12]. These findings are stated to be regardless of pretreatment N-staging. However, it was found that 56% of neck dissections completed did contain residual disease, including a patient who was thought to have complete response based on CT imaging [9]. Clinical response based on physical examination, on the other hand, has been found to be a poor indicator of residual neck disease [12, 13]. These groups, and others, agree that incomplete or partial radiographic response definitely warrants postradiotherapy neck dissection, identifying significantly lower survival rates in those with incomplete radiographic response who did not undergo surgery [9, 10, 14]. The predictive value of radiographic clinical response cited by these groups remains controversial, as Brizel et al., in their study of 154 patients, found the negative predictive value of clinical complete response at only 74% [3]. And yet more groups remain divided down the middle, stating clinical complete response to be an adequate indication for observation in N2 disease, but inadequate in cases of N3 neck disease [4, 15].

 There continues to be concern about the rates of residual microscopic neck disease in the post-RT N2/N3 neck, regardless of clinical response. It is important to note that the rate of morbidity and complications in planned post-RT neck dissection has been shown not to be significantly different from those of radiotherapy alone [16, 17]. Furthermore, salvage surgery, when required, has a significantly lower likelihood of success compared to definitive initial treatment of disease, and recurrent neck disease results in an extremely poor quality of life [18, 19]. Definitive cure with the initial treatment is therefore critical in the overall management of neck metastasis in head and neck cancer. In 51 neck dissections of postchemoradiation patients with N2 or greater disease, Kutler et al. experienced only one neck recurrence despite 32% of specimens showing residual carcinoma [20]. In another study, 53 N2-3 patients were divided into those who received PPRND and those who did not. Of the 35 who underwent neck dissection, only one patient had neck recurrence, while of the 30 who did not, 9 experienced recurrence and were not successfully salvaged. Of the 30 patients who were thought to have clinical complete response, 7 had persistent or recurrent disease [21].

 For centres with PET scanners, the controversy of planned postradiotherapy neck dissection has been put to rest. In 2007, Yao et al. published their findings of PET as a predictor of residual cervical lymph nodes in patients with head and neck squamous cell carcinoma following radiotherapy. They found a positive predictive value of 62.5% and a negative predictive value of 100% [22]. Where available, a negative PET scan can be used to settle to controversy of planned postradiotherapy neck dissection; however, many centres still do not have access to PET technology and must rely on the published literature to guide their dissection. 

 Our results showed that 31% of planned postradiotherapy neck dissection showed evidence of residual carcinoma, which is in keeping with previous literature [20, 21]. Psychogios et al. have shown that advanced head and neck cancer (pT3-4) in general has consistently shown occult metastasis in 24.5–53.3% of clinically negative specimens [23]. Patients with disease-positive dissections are more likely to have failed neck disease control, as we observed that 44% of positive-specimen patients had recurrence, compared to 17% of negative-specimen patients (*P* = 0.066). Failed disease control in the neck correlates with a statistically significant decrease in survival, when compared to patients without recurrence (*P* = 0.01) and all advanced head and neck squamous cell carcinoma patients regardless of recurrence (*P* = 0.05). These findings are especially apparent in diagnosed with ≥N2 disease.

## 5. Conclusion

Achieving successful long-term outcomes in patients with metastatic head and neck cancer remains a significant therapeutic challenge, particularly in cases of advanced disease on presentation. Completing a planned neck dissection following primary radiotherapy has been a controversial management topic, prior to the introduction of positron emission tomography (PET) for establishing postradiation positive disease. For centres that still do not have access to PET scanners, the necessity of performing a planned post-radiotherapy neck dissection continues to be questioned. Based on the frequency of residual malignancy in the neck of patients treated with primary radiotherapy, a planned, postradiotherapy neck dissection should be strongly advocated for all patients presenting with advanced-(N2-3) stage neck disease.

## 6. Summary

- Optimal therapy for patients with head and neck cancer with metastatic neck disease remains both controversial and challenging.
- Radiation alone or in conjunction with neck dissection produces equivalent neck control rates for early-stage disease (N0/N1).
- Treatment in the advanced head and neck cancer patient (N2-N3) remains more controversial.
- Some feel that clinical and radiographic response is sufficient to guide the use of postradiotherapy neck dissection.
- Some advocate for uniform neck dissection, based on high rates of persistent microscopic disease following radiotherapy.
- For centres with PET scanners, use of this technology is sufficient to predict residual disease and guide the use of neck dissection.
- Our group has found that 31% of postradiotherapy patients have residual disease, leading to significantly greater failed neck control and a significant decrease in survival.
- This study adds to the current literature supporting the use of uniform post-radiotherapy neck dissection and should contribute to settling the controversy of management in advanced head and neck cancer in centers without PET scan technology.

## Figures and Tables

**Table 1:** Distribution of primary malignancy site.

	*n* = 28	(%)
Location of primary		
Tonsil	13	(46)
Tongue base	10	(36)
Piriform sinus	2	(7)
Glottis	1	(4)
Unknown primary	2	(7)

**Table 2:** Primary staging and nodal status.

	*n* = 28	(%)
Primary tumor		
T1	2	7
T2	11	38
T3	8	29
T4	5	18
Tx	2	11
Neck disease		
N0	2	7
N1	3	11
N2a	6	21
N2b	8	29
N2c	6	21
N3	3	11

**Table 3:** Pathology results of neck dissection specimens.

	Specimens collected	Carcinoma present	%
Neck disease			
N0	2	1	50
N1	3	0	0
N2a	6	2	33
N2b	8	4	50
N2c	6	3	33
N3	3	0	0
